# A Chemometric-Assisted Colorimetric-Based Inexpensive Paper Biosensor for Glucose Detection

**DOI:** 10.3390/bios12111008

**Published:** 2022-11-11

**Authors:** Vinay Kishnani, Shrishti Kumari, Ankur Gupta

**Affiliations:** Department of Mechanical Engineering, Indian Institute of Technology, Jodhpur 342037, India

**Keywords:** chemometric detection, glucose, smartphone-based sensors, machine learning

## Abstract

This article reports a simple and inexpensive leak-proof paper pad with an initial selection of a paper substrate on the grounds of surface morphology and fluid absorption time. Herein, a drying method is used for glucose detection on a paper pad through colorimetric analysis, and the spot detection of glucose is analyzed by optimizing the HRP concentration and volume to obtain accurate results. The rapid colorimetric method for the detection of glucose on the paper pad was developed with a limit of detection (LOD) of 2.92 mmol L^−1^. Furthermore, the effects of the detection conditions were investigated and discussed comprehensively with the help of chemometric methods. Paper pads were developed for glucose detection with a range of 0.5–20 mM (apropos to the normal glucose level in the human body) and 0.1–0.5 M (to test the excessive intake of glucose). The developed concept has huge potential in the healthcare sector, and its extension could be envisioned to develop the reported paper pad as a point-of-care testing device for the initial screening of a variety of diseases.

## 1. Introduction

The first thought in the anticipation and treatment of disease is a meticulous diagnosis, but diagnostic technologies which are efficient in the economically industrialized world are often hard to avail in countless inaccessible places. It is expensive for common people to afford the examinations. As per the world health organization (WHO), diagnostic devices for remote locations in developing and underdeveloped countries should be affordable, sensitive, specific, user-friendly, rapid, robust, equipment-free, and deliverable (ASSURED) to the end-users. Since glucose metabolism is essential to life, detecting the human body’s glucose level is one of the most frequently performed tests in hospitals and laboratories [1]. Hypoglycamia and diabetes mellitus are medical conditions related to irregular glucose metabolism. According to the international diabetes federation (IDF), diabetes will affect approximately 643 million people globally by 2030, rising to 783 million by 2045 [2]. Early diabetes symptoms are still not clear. It could lead to severe complications, such as neuropathy, cardiovascular disease, and end-stage renal disease, by missing the proper diabetes management time [3]. Therefore, creating a sensor that allows an adult to diagnose his or her diabetes quickly is crucial to address this unmet healthcare need. Along with micro-electro-mechanical system (MEMS) based drug delivery devices, flexible sensors are also playing a crucial role in the area of biomedical applications. Flexible sensors with wireless sensing modules are being developed in the biomedical field for the ease of end-user purposes [4,5,6].

Previously, Müller and Clegg reported the first kind of paper-based microfluidic device in 1949 [7]. However, Whitesides’ group later explored paper-based microfluidics and opened new pathways in this field for the use of papers to develop portable, on-site detection in bio-sensing applications [8].

These paper-based analytical devices (PADs) are primarily paper substrates with hydrophilic channels and hydrophobic barriers all around, created via stamping, photolithography, wax printing, and dipping, among many other fabrication techniques [9]. PADs combine capillary management for the transportation of fluid, limited sample sizes, and minimum usage of reagents with the benefits of a paper platform, such as ease of availability, cost-effectiveness, bio-compatibility, flexibility, a light-weight quality, fluid movement without external pumps, and hydrophilicity [10,11,12]. Bio-sensing can be performed via a dipstick model [13], spot-based, and lateral flow analysis. Furthermore, electrochemical and colorimetric methods are the most frequently used detection techniques for the analysis of paper devices due to their portable, low-cost, simple, and easy-to-use instrumentation. Additionally, for more specific and enhanced colorimetric and electrochemical analyses, μPADs are reported to be modified through nano-functionalized materials [14,15,16] and biopolymers viz. chitosan [17]. The development of an advanced point-of-care testing device, which provides fast, accurate, and specific data, needs automation, structural data collection, planning, and monitoring which could further be acquired through the internet of things (IoT) [18]. Paper-based sensors may capture color information in a variety of color schemes, including RGB (red, green, and blue), HSV (hue, saturation, and value), and L*a*b* (lightness, green-red, and blue-yellow) [19,20,21,22]. A number of articles have reported methods for applying different color spaces for the detection of various chemicals. For instance, the detection of textile dye (BR9) and alcohol content in saliva were both performed using the HSV color space, which was transformed from RGB [23,24]. In comparison, sensitive pH measurements between 1 and 12 were performed using the L*a*b* color space [25]. For the purpose of detecting chlorine in water [26] and estimating the freshness of fruits [27], the RGB color space was used directly. The colorimetric analysis was carried out using an analytical expression-based method to investigate the properties of the color space. Unfortunately, light sources and camera optics have a significant impact on colorimetric analysis. To eliminate the effects of light and increase the detection threshold, researchers employed 3D-printed light boxes in the field of colorimetric sensors [28,29]. In the quantitative evaluation process, sophisticated algorithms such as machine learning were suggested as a solution to this problem [30]. Machine learning, which has useful features such as automated decision-making and self-learning from data, has become popular in the field of data analysis. The advantages of such tools in biosensors includes the opportunity of attaining realistic methodical outcomes from noisy, undesirable, and low-resolution sensing statistics that could comprehensively coincide with each other. Additionally, the proper deployment of machine-learning (ML) methods could realize unknown relations between sample constraints and sensing signals through data visualization. In particular, ML could be applied in a variety of ways to study the raw sensing data from a biosensor. Numerous systems executing complex algorithms have been created that conduct colorimetric analyses for trustworthy qualitative and quantitative assessments of colorimetric assays, thanks to recent advancements in smartphone technology. As an example, to detect nitrite in food, a mobile phone app was reported that combined a polyacrylonitrile nanosheets (PAN)-NSS color sensor and a deep convolutional neural network (DCNN) for fast sampling, transmission, and data processing [31]. A smartphone-based lateral flow imaging system was developed by using ML classifiers for the detection of *salmonella* spp., a foodborne pathogen. Multiple models were used for the training of the data set, and it was found that support vector machines (SVMs) and k-nearest neighbors (KNNs) produced the highest accuracies, up to 95.56% [32]. To detect glucose in artificial saliva, a machine learning-based smartphone app was developed. This platform was classified on the basis of a color change under different illumination conditions and different smartphones [33]. In another work for the detection of pH, glucose, and protein in the urine, a colorimetric reader application was developed with inter-phone repeatability [34]. In spite of increasing admiration, smartphone-based colorimetric analyses have some issues, including simplicity, reliability, and accessories. It is necessary that the obtained results from smartphones should be accurate in any situation. The obtained results could deviate under different environmental conditions and accessories, viz., illumination, different phones, etc. The results are also sensitive towards the camera optics and smartphone [33,34,35,36]. 

The expected range for normal fasting blood glucose levels is 3.9 mM to 5.6 mM or somewhere in between. Changes in lifestyles and the monitoring of glycemia are advised when fasting blood glucose levels are between 5.6 and 6.9 mM. The normal level of glucose is found within a limit of 2.5–5.3 mM in serum and 0.1–0.8 mM in urine. In this work, we demonstrated the colorimetric analysis of glucose on a paper pad, and machine learning was used for the optimization of the color variation. 

## 2. Experimental Procedure

### 2.1. Materials and Instrumentation

The following were utilized for experimentation: Whatman no. 1 and no. 4 filter paper (125 mm diameter) from GE Healthcare Life Sciences, permanent marker (Camlin, local vendor), Ultra Gold reagent grade Triple Deionized water (DIW) from Organo Laboratories Chemical Pvt Ltd., Delhi, India (specific conductivity < 1), Glycerol from Sigma-Aldrich (St. Louis, MO, USA) (≥99.0%), Methylene Blue (MB) dye from Fisher Scientific (Waltham, MA, USA) (Sp. Absorptivity A 1%/1 cm max at 660–665 nm), glucose oxidase (GOx) from Aspergillus niger Type X-S, lyophilized powder, 100,000–250,000 units/g solid, Potassium Iodide (KI) from Sigma-Aldrich(ACS reagent ≥99.0%), Horseradish Peroxidase (HRP) from Sigma Aldrich, Type VI, $\left(\geq 250\frac{\mathrm{units}}{\mathrm{mg}}\;\right)$(solubility: 0.1 M PBS soluble 10 mg/mL), Trehalose from Molychem, Mumbai, India (minimum assay- 99%), D-Glucose from Thermo Fisher Scientific Private Limited, India, and phosphate-buffered saline (PBS) from Sigma Aldrich, Water Soluble, pH: 7.2–7.6 (1 tablet/200 mL), HCl purchased from Sigma Aldrich India, Smartphone (Realme 8), scanning electron microscopy (SEM-Carl Zeiss-EVO 18, EDS-Oxford instruments-51-ADDD-0048, Oxford Instruments, Cambridge, UK), Atomic force microscopy (Park System-XE70, Park System, Seoul, Korea), contact angle goniometer (KRUSS GmbH-DSA25B, KRUSS, Hamburg, Germany) equipped with ADVANCE Software, printer (HP LaserJet M1005, HP, Delhi, India), and a commercially available laminator. Unless otherwise noted, all chemicals were acquired at the highest analytical grade and used without any purification.

### 2.2. Selection of Paper as a Base Substrate

**Figure 1:**
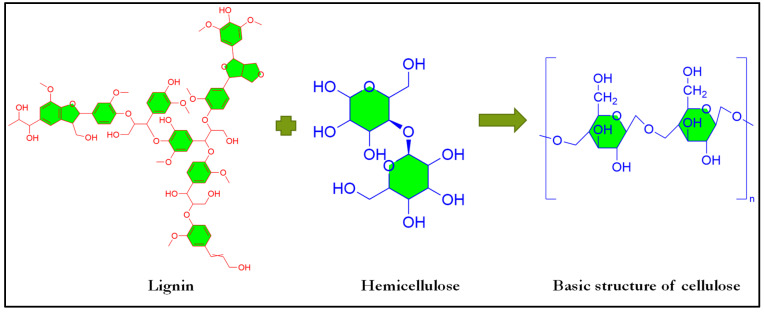
Schematic of the basic structural composition of cellulose.

Paper, in general, is composed of cellulose fibers, and basic cellulose is composed of lignin and hemicellulose, as shown in Figure 1. However, due to variations in material compositions and production methods, each variety of paper has unique mechanical characteristics. Given that different forms of paper have distinct components, it is challenging to generalize a set of attributes for paper. Based on its content and structure, each variety of paper performs a certain purpose differently. Cellulose fiber in printing paper is combined with a sizable quantity of filler material. Natural minerals like clay, talc, and limestone, as well as synthetic substitutes, viz., gypsum, precipitated calcium carbonate, and titanium dioxide, could be used as filler material. The composition, quality, and type of the filler ingredients determine the paper’s appearance, thickness, and structure. The filler determines the paper’s production costs, strength, brightness, and refractive index, as well as the energy needed for drying and friction, the burn rate, and the pore size. Cellulose is found in complex lignocellulose composites that are primarily composed of lignin and hemicellulose. Fillers could have a detrimental impact on the sheet’s two-sidedness, durability, endurance, and abrasion resistance.

For the selection of the right paper for the paper-based biosensors, thirteen different paper substrates were collected and investigated using the drop analysis technique. A 0.5% MB dye solution was used for the drop analysis. The thirteen different substrates that were investigated were: Whatman no. 1 paper, bond paper, craft paper (200GSM), handmade blotting paper, tissue paper (2 ply), glossy paper, card sheet paper, practical sheet paper, ivory sheet paper, sketch sheet paper, drawing sheet paper, A4 paper, and Whatman no. 4 paper. A drop of 10 µL of methylene blue (MB) dye solution was dropped onto the paper surface through a micropipette, and the time taken for the dye to spread and absorb on the paper surface was recorded using a stop-watch. Different paper substrates have different surface morphologies and pore sizes, which are responsible for the different absorption times.

**Figure 2:**
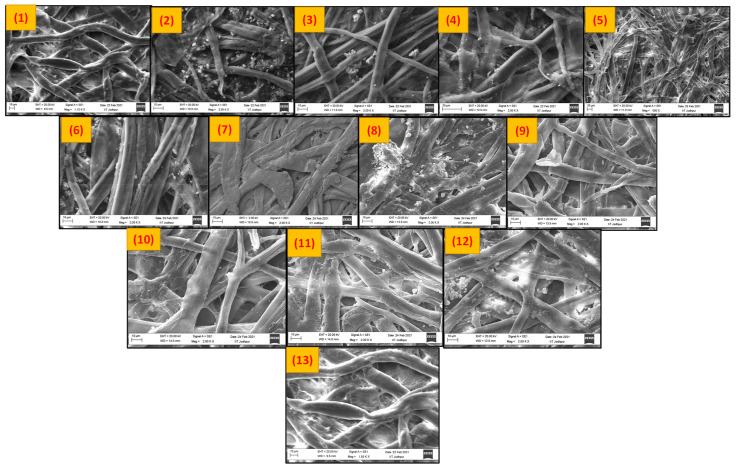
SEM images of different types of papers available: (1) Whatman no. 1, (2) bond paper, (3) 200 GSM card sheet, (4) blotting paper, (5) paper towel (2 ply), (6) glossy paper, (7) 300 GSM card sheet, (8) practical sheet paper, (9) ivory sheet, (10) sketch sheet, (11) drawing sheet, (12) A 4 paper (67 GSM), and (13) Whatman no. 4.

Figure 2 shows the SEM images of the thirteen commercial papers available on the market. It was found that most of the commercial paper contained foreign metals, viz., Si, Al, Ca, Mg, and Cl, apart from cellulose (Table S1). These foreign materials are added to the paper pulp as a filler material in alkaline papers to increase the brightness and opacity. It is hard to create offset lithography in papers due to the availability of calcium carbonate. The papers could also include trace levels of magnesium carbonate, silicon dioxide, aluminum oxide, and iron oxide in addition to calcium carbonate. These filler materials also clog the pores of the paper substrate, which affect the porosity, permeability, and wicking properties of the paper.

**Figure 3:**
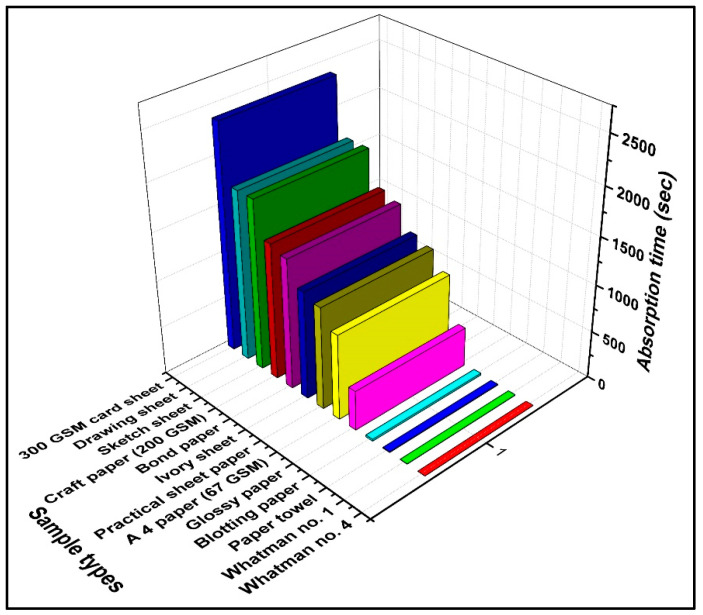
Graph representing the absorption times for a fixed volume of dye solution with Sdifferent papers.

Whatman filter paper was selected for further analysis because it showed good holding capacity and the lowest absorption time compared to other papers, as shown in Figure 3. The commercial A4 paper available on the market contained some foreign materials, as shown in Figure 2 (12), which was confirmed with the EDX analysis, through which it was determined that it contained C, O, Ca, and Cl (Table S1). These foreign materials blocked the pores of the A4 paper, which resulted in absorption time enhancement, as shown in Figure 3.

### 2.3. Characterization Techniques

The surface morphology of the permanent ink-modified surface and the unmodified surface of the paper pad was analyzed through atomic force microscopy (AFM) using an advanced scanning probe microscope (Park Systems XE-70, Suwon, Korea). The data gain was set at −144.74 × 10^−6^ (µm/step), I Gain-1, and P Gain-1. Scanning electron microscope (SEM) (ZEISS and OXFORD instrument) images were obtained. Preceding the SEM readings, the samples were treated with a thin layer of gold to avoid and diminish charging on the surface. All contact angle (CA) measurements were performed with a goniometer (KRUSS GmbH model no. DSA25B equipped with ADVANCE Software). For the static water CA measurements, DI water was dropped onto the sample surface, and the CA was measured at a relative humidity of 40 ± 5% and a temperature of 20 °C. A piece of filter paper with a dimension of 10 × 10 mm^2^ modified with hydrophobic ink was taken for the contact angle measurement. There was no backing of tape or any other material to determine the exact contact angle generated with the hydrophobic ink. A droplet of a 0.47 mm mean diameter and a 0.045 µL mean volume (automatically calculated by the instrument software) was dropped over the modified surface. The contact angle was calculated after the generation of the static angle and was determined up to 108.69 ± 0.25°.

### 2.4. Fabrication of the Paper-Based Sensor and Colorimetric Detection

**Figure 4:**
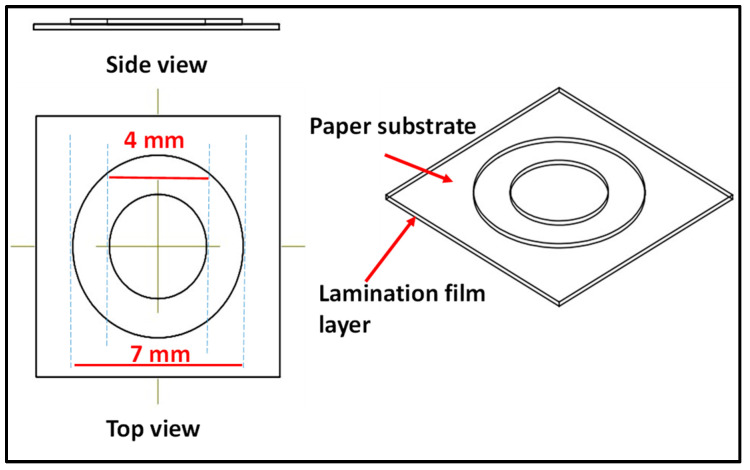
Schematic representation of the dimensions and design of the paper pad.

Design patterns were drawn with SOLID WORKS (2020). The design of the microfluidic paper pad consisted of two concentric circles having sample zones of 4 mm. The area between the outer periphery of the inner circle and the inner periphery of the outer circle was used as a boundary to create a hydrophobic barrier, as shown in Figure 4. The designs were printed on Whatman paper with an HP printer using a black toner cartridge from HP LaserJet M1005. The back side of the sensing zone was covered with laminating film. Then, the area between the two concentric circles became hydrophobic, while the center area remained hydrophilic as a testing zone. The patterned paper was then placed in a vacuum desiccator for drying the paper pad, as well as for the prevention of atmospheric contact for further use.

### 2.5. Reagent Preparation

For the glucose assay, two different concentration ranges were chosen; the first was 0.1–0.5 M with a difference of 0.1 M for the color development, and the other was 0.5–20 mM. The reason for selecting these values was that normal blood glucose levels lie in this range. To obtain both concentration ranges, glucose was dissolved in PBS (0.1 M, pH 6.0). KI, glucose oxidase, and HRP were dissolved in PBS (0.1 M, pH 6.0) to obtain 0.6 M, 120 U/mL, and 100 U/mL of stock solution. The prepared stock solution was kept in a refrigerator at 4 °C, ceasing from the light, while the KI was stored at room temperature, wrapped with aluminum foil to protect from light for further use.

**Figure 5:**
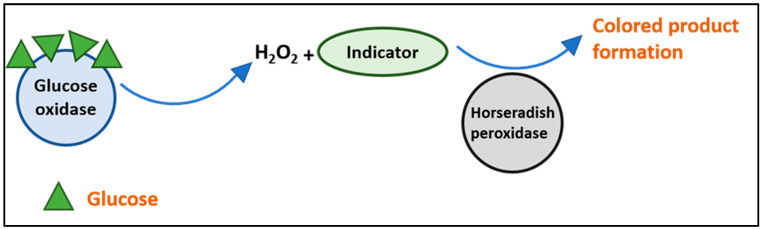
Schematic representation of the reaction mechanism for the colored product formation using oxidase enzymes.

The colorimetric detection of glucose is based on oxidase enzyme activity. Glucose oxidase is specific for glucose molecules; it binds and converts it into gluconic acid, and thereafter, hydrogen peroxide is released. The hydrogen peroxide then combines with an indicator, here KI, and converts the iodide from potassium iodide to triiodide in the presence of horseradish peroxidase, resulting in the formation of a colored product, as shown in Figure 5.

### 2.6. Optimization of the Volume and Concentration

**Figure 6:**
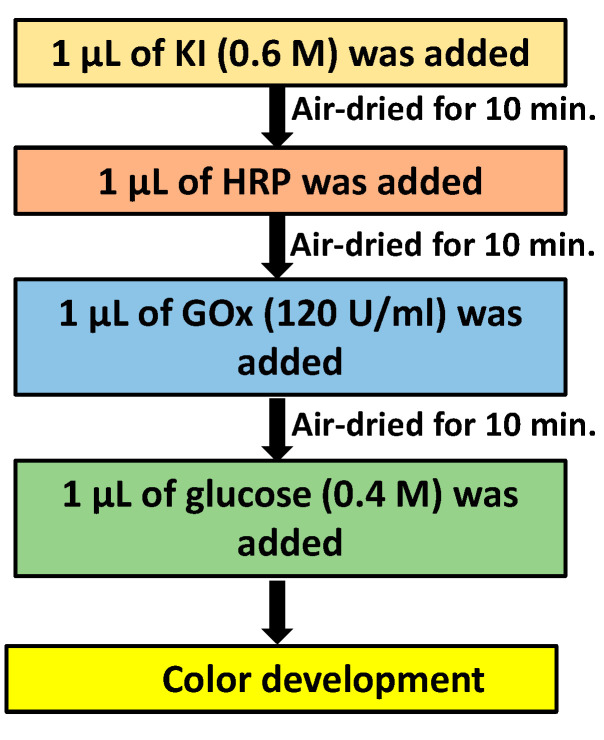
Schematic showing the reaction mechanism for color development using different concentrations of HRP.

The sample and reagent volume optimization was performed using a solution of MB dye and glycerol on the spots on Whatman no. 1 and no. 4 filter papers to determine the minimum volume of the sample and reagents required to fill the zone completely. For the optimization of HRP, different concentrations, i.e., 10 U/mL, 20 U/mL, 30 U/mL, 40 U/mL, 50 U/mL, 60 U/mL, 70 U/mL, 80 U/mL, 90 U/mL, and 100 U/mL were prepared. The steps for the optimization of the different concentrations of HRP for the detection of glucose are shown in Figure 6.

**Figure 7:**
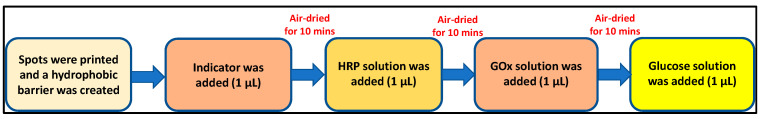
Schematic showing the reaction mechanism for glucose detection.

For the detection of glucose, the indicator (potassium iodide) was first added to the detection pad, and then it was air-dried for 10 min, followed by the addition of horseradish peroxidase, after which it was air-dried for 10 min. After the indicator and HRP were completely dried at room temperature, glucose oxidase was added and air-dried for 10 min. After completing the process, the detection pad was ready for testing and was stored in a refrigerator at 4 °C for further use. The final test was conducted by adding different concentrations of glucose to the different spots (Figure 7).

### 2.7. Method of the Digitization of the Obtained Result

The raw images obtained from the smartphones (Realme 8) were then pre-processed to progress to further steps, which included feature extraction and machine learning modeling. The images were pre-processed to obtain the actual region of interest (ROI) where the main color change took place. Various noise reduction techniques were used to obtain the ROI. After that, a mask was created, which was placed over the actual resized image to obtain the ROI. The images were converted to various color spaces or gamuts available in MATLAB to obtain the maximum possible derived feature vectors.

The color spaces used included HSV, L*a*b*, XYZ, gray-scale (the scale varied from 0–255, in which 0 stood for white and 255 stood for black, and the in-between gray shades were obtained), and NTSC. The NTSC images had three attributes, as shown in Table 1.

Where HSV stands for hue, saturation, and value, L*a*b* stands for perceptual lightness (L), and a and b for the four unique colors of human vision. In comparison, XYZ stands for (an X-mix of RGB curves treated as non-negatives, Y-luminance, and a Z-quasi equal to blue).

## 3. Results and Discussion

### 3.1. Sample and Reagent Volume Optimization

It is known that normal blood viscosity lies within the range of 3.5 cP to 5.5 cP. Therefore, in order to mimic blood spreading in the paper-based spot detection, 40% glycerol was used, which consisted of a viscosity of 3.7 cP, lying well within the range of normal blood viscosity. A 0.2% methylene blue dye solution was prepared in 40% glycerol. The reasons for choosing methylene blue were its properties, viz., its antioxidant, cationic, and organic nature, ease of solubility in water and glycerol, etc., and its bright color for ease of detection. The optimized volume of the dye solution, which covered the entire testing zone, was found to be 1 µL and 1.5 µL for Whatman no. 4 and Whatman no. 1 filter paper, respectively (Figure S1). 

### 3.2. Characterization of the Detection Pad

**Figure 8:**
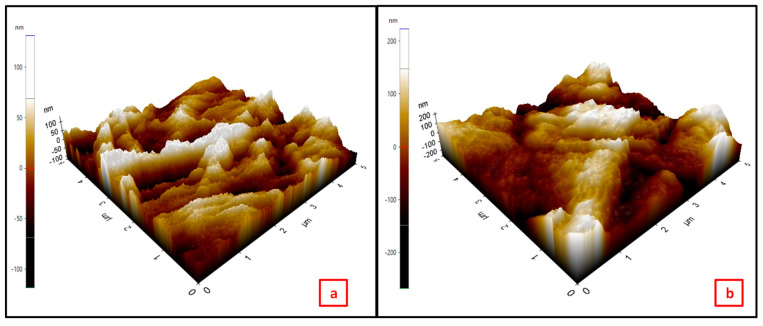
AFM images of the (**a**) unmodified surface and (**b**) ink-modified surface of Whatman no. 4 paper.

Atomic force microscopy images of the permanent ink-modified surface and the unmodified surface of the detection pad revealed that the roughness of the modified surface increased slightly in comparison to the unmodified surface. However, the surface of the modified paper appeared to be smoother in comparison to the unmodified surface, as shown in Figure 8. The root mean square roughness factor for the unmodified and modified surface was calculated to be 35.160 nm and 75.449 nm, respectively. The unmodified surface of the paper showed a rough contour of the fibrous structure, while the ink-modified surface appeared smooth, as the ink absorbed over the fibrous surface and filled the pores of the paper surface. If the surface were rough instead of flat, the actual wet surface area would be greater in comparison to the geometric area. As a result, a net increase in the surface energy means that the surface would be more hydrophobic. Cross-sectional images of Whatman paper no. 4 are shown in Figure 8. 

**Figure 9:**
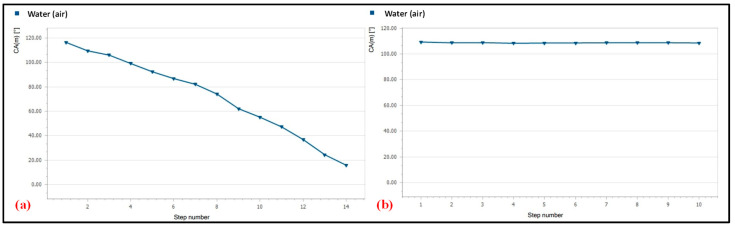
Contact angle retention after an (**a**) single-stroke or (**b**) double-stroke.

For the analysis of the hydrophobicity, the paper was coated with a single-stroke and double-stroke of hydrophobic ink. To determine the contact angle over the modified paper surface, a sessile drop test was performed with a contact angle goniometer (DSA25B) at a mean temperature of 20.0 °C for a mean volume of 0.045 µL of DI water. It was observed that a single stroke of hydrophobic ink was not sufficient for holding the water droplet because it decreased continuously. In comparison, using a double stroke of hydrophobic ink held the water properly for a longer time, as shown in Figure 9.

**Figure 10:**
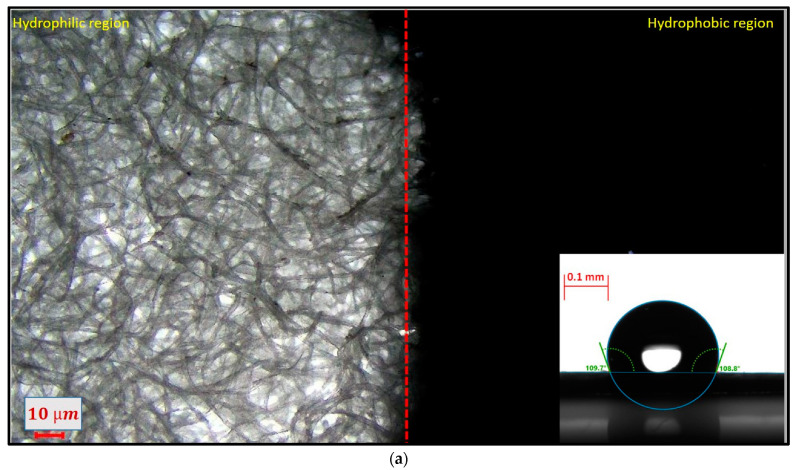

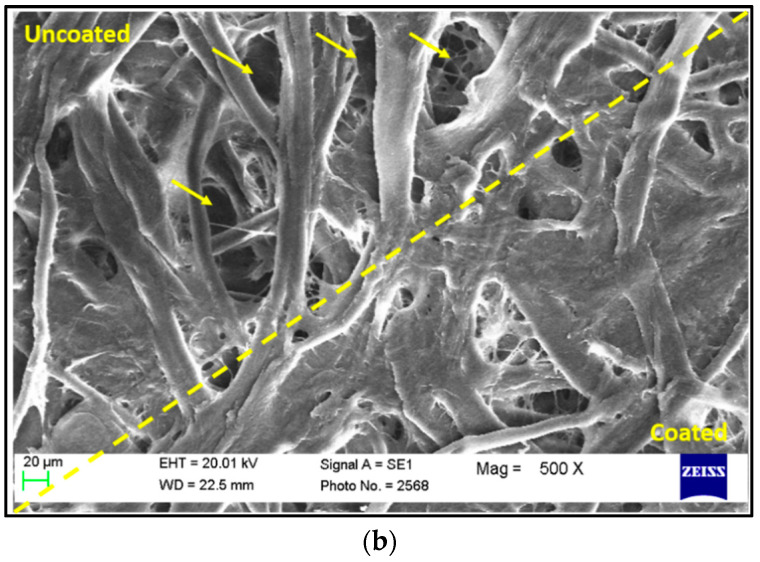
(**a**) Microscopic image of the junction of the unmodified and modified paper (inset contact angle of the modified paper). (**b**). SEM image of the junction of the unmodified and modified paper.

Figure 10a shows the microscopic image of the ink-modified surface of Whatman no. 4 paper, and the red dotted line shows the boundary between the modified and unmodified surfaces. The mean contact angle of the double-stroke coating was observed up to 108.69 ± 0.25° (bottom right-most corner of Figure 10a).

Figure 10b shows the SEM image of the double-stroke hydrophobic ink-coated paper substrate. It was observed that the hydrophobic ink partially blocked the pores of the paper substrate, while the unmodified surface had deep and wide pores in comparison to the modified paper.

### 3.3. Optimization of the Concentration

**Figure 11:**
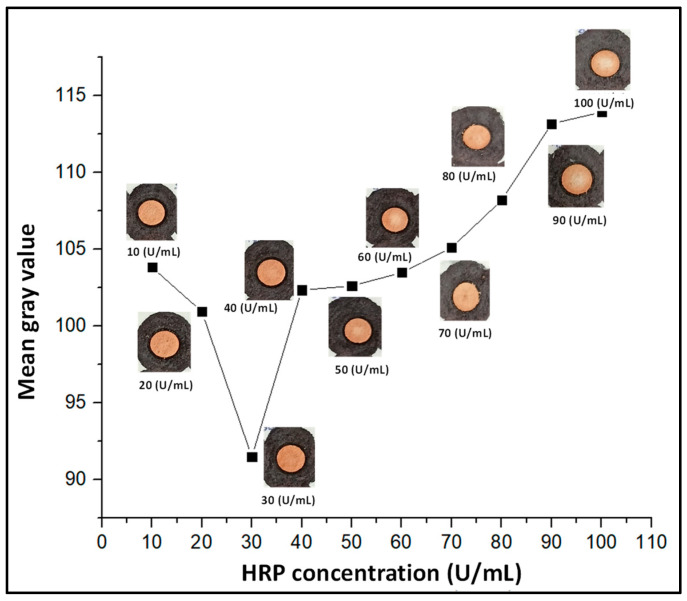
Graph showing the mean gray value for different concentrations of HRP.

Furthermore, the HRP concentration optimization was performed for the range of 10–100 U/mL. It was observed that initially, the mean gray value decreased until 30 U/mL, but after 30 U/mL, it rose sharply. The lowest mean gray value indicated that the darkest shade was obtained at 30 U/mL of HRP, while an increase in the mean gray value meant a lighter shade. This could be due to an increased concentration of enzyme for the fixed concentration over the substrate, as no additional substrate was available to bind to the enzyme. Figure 11 shows the optimization curve for HRP for a range of 10–100 U/mL.

### 3.4. Glucose Detection

**Figure 12:**
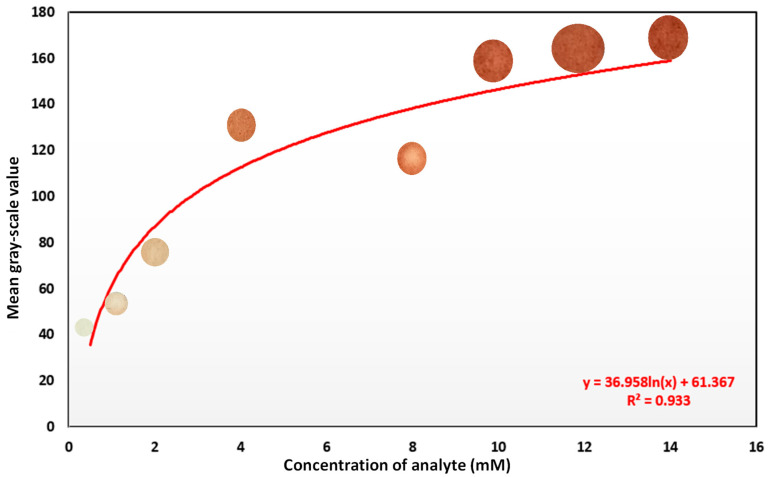
Response curve of the spot-based glucose assay. The response curve is represented with the solid line and was obtained from a non-linear curve fit of y=36.958∗ln(x)+61.367 with an $R^{2}=0.933$.

Color developments were observed, and images were captured using the smartphone (Figure S2). Images of the detection pad were taken via a Realme 8 smartphone in the jpeg format with the following specifications: 64 MP, aperture: f/1.8, sensor size: 1/1.73”, pixel size: 0.8 μm, focal length: 26 mm, and image stabilization: PDAF (phase detection auto-focus). The images were taken in an open ambient light without flashing homogeneously. These images (Figure S3) were taken for different concentrations, two of which were of unknown concentrations. The raw images were then pre-processed to progress to further processes, including feature extraction and machine learning modeling. Images were pre-processed to obtain the actual region of interest (ROI) where the main color change took place. Various noise reduction techniques were used to obtain the ROI. After that, a mask was created, which was placed over the actual resized image to obtain the ROI. A response curve of the glucose assay (Figure 12) was constructed. It showed a non-linear curve with an $R^{2}=0.933$, and the image intensity was stable up to a 14 mM concentration. The image intensity was unstable above this range. 

### 3.5. Digitization of the Obtained Results

The classification learners in MATLAB (R2021b) were used to assess the performance. The training data were analyzed by creating the training model using supervised learning. Support vector machines (SVMs) were used in MATLAB to discover the decision boundary that would best divide the classes and maximize the margin. SVMs are used to search for the hyperplane that maximizes the margin in the linearly separable scenario, provided that both classes are correctly identified. Soft Margin and Kernel Tricks are two principles that SVMs use to solve non-linearly separable scenarios. Kernel changes the dimensionality of the dataset if the dataset is not separable in the lower dimensions. Statistical feature vectors were obtained from all the color spaces, which included the mean (Figure S4), skewness (measurement of the asymmetry of the probability distribution) (Figure S5), and kurtosis (its value defines the shape of the probability distribution). Figure S6 created 45 features. The gray-level co-occurrence matrix (GLCM) functions to characterize the texture of an image by calculating the frequency of the pairs of pixels with particular values and in a specified spatial relationship, creating a GLCM, and then retrieving the quantifiable metrics from this matrix, including contrast, correlation, energy, homogeneity, entropy, and intensity of the gray-scale (Figure S7). All of these features constituted a total of 51 features for each image.

**Figure 13:**
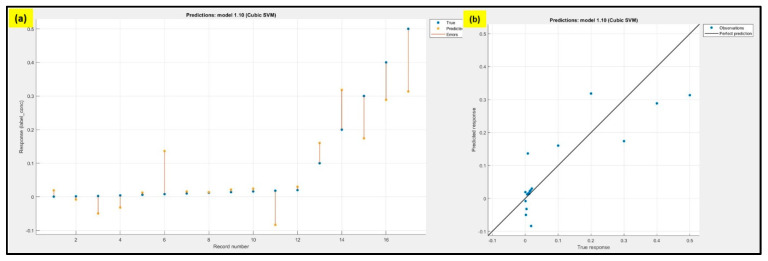
(**a**) Errors in prediction with respect to the actual data, (**b**) the predicted vs. actual data.

Figure 13a shows the error in the prediction with respect to the actual data value, where the blue dots represent the actual data, the orange dots represent the predicted data value, and the line between them shows the error in the data, while Figure 13b shows the best-fit graph between the predicted and actual data. The R square value was 0.74. Further trained data were tested for two unknown samples (Figure S8), and the predicted concentrations were 7.18 mM and 0.35 M.

### 3.6. Shelf-Life Testing

Shelf life testing was also performed after the immobilization of the enzymes (HRP and glucose oxidase) and the reagents on the paper. Trehalose was added in one set that was kept at room temperature and one kept at 4 °C. A comparison was performed between the detection pad kept at room temperature and 4 °C for 14 days. Shelf life testing was conducted to determine the performance and lifetime of the pad. The color appeared to be darker for the spots in which trehalose was added, as it maintained the enzyme stability (Figure S9). Different spots were tested at different intervals of days using a glucose solution of a fixed concentration. The shade or darkness of the color decreased as the number of days increased. Images of the color were developed after the addition of the glucose solution at different numbers of days and were captured using a smartphone for all four sets, and the mean gray value was analyzed using MATLAB. 

**Figure 14:**
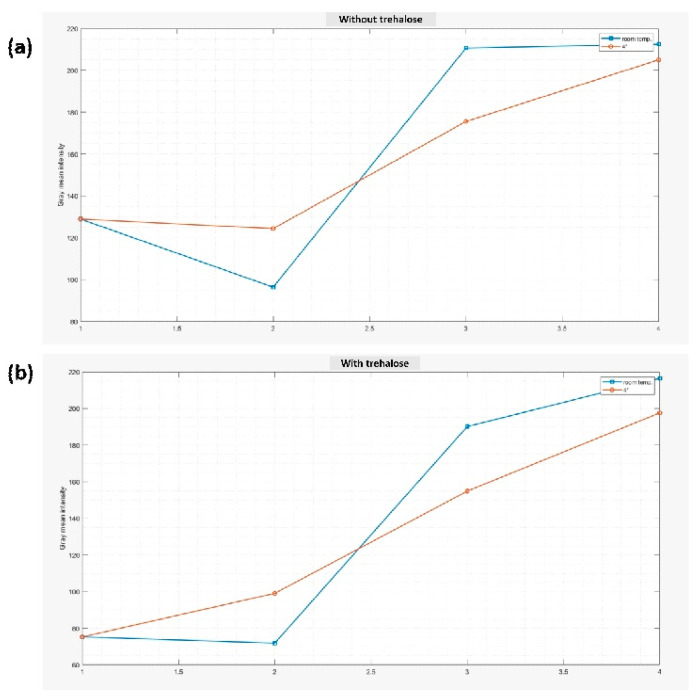
Graph showing the mean gray values for the set (**a**) in which trehalose was added (**b**) in which trehalose was not added and kept at room temperature and 4 °C.

Figure 14 shows the mean gray value, which first decreases and then increases with the number of days, which indicates a lighter shade of color developed. The mean gray value on the 14th day was lower for the one which was stored at 4 °C and had trehalose immobilized. However, the color developed for this spot was also lighter as compared to the first day and showed approximately 62% decreased color intensity. It could be stored at 4 °C, but the result would not be accurate if a surface modification were not conducted. 

## 4. Conclusions

In this work, paper as an inexpensive and potential substrate material for point-of-care testing was investigated under ASSURED criteria. Herein, colorimetric analyses were performed through machine learning techniques in a controlled environment for the detection of glucose on a leak-proof pad, which was fabricated by creating a hydrophobic zone through the proper penetration of ink into the pores of the paper. A detailed analysis was carried out for the ink-coated paper zone through the surface roughness (75.449 nm) analysis and contact angle (108.69 ± 0.25°) measurement. Further, the fabricated detection pad was tested for the optimization of samples, and the determination of glucose for different concentrations with a LOD of 2.92 mmol L^−1^. Furthermore, machine learning classifiers were implemented for the color-change analysis in µPAD for the detection of glucose levels in the samples. These classifiers were trained using images captured through smartphones under ambient lighting conditions. Trained data were tested for the two unknown concentrations. The obtained R square value for the trained data was 0.74, and the predicted concentrations were 7.18 mM and 0.35 M. To further improve the accuracy, work was also performed on increasing the dataset for the determination of closer concentration levels. It could be concluded that the fabricated biosensor platform with the explored chemometrics is economical, robust, and could be fabricated easily without the use of any heavy instrumentations, and the generated machine learning platform could be helpful in the statistical analysis for the detection of biological samples.

## Figures and Tables

**Table 1:** The description of the attributes of the images.

Attribute	Description
Y	Luma, also known as picture brightness. Values fall between [0, 1], where [0] designates black and [1] designates white. As Y grows, colors get brighter.
I	The proportion of blue or orange tones in the picture is known as in-phase. The values of “I” fall between [−0.5959, 0.5959], where a negative number represents a blue tone, and a positive number represents an orange tone. The intensity of the color grows as I’s magnitude rises.
Q	The proportion of green or purple tones in the picture is known as quadrature. Q contains a value of between (−0.5229) and (0.5229), where (−) denotes a green tone and (+) denotes a purple tone. The intensity of the color grows as Q’s magnitude rises.

## Data Availability

Data will be provided based on the request.

## Supplementary Materials

The following supporting information can be downloaded at: https://www.mdpi.com/article/10.3390/bios12111008/s1, Table S1: EDX analysis of papers; Figure S1: Sample and reagent volume optimization using MB dye solution; Figure S2: Contact angle measurement of single stroke coated ink over Whatman.4 paper; Figure S3: Contact angle measurement of double stroke coated ink over Whatman.4 paper; Figure S4: Colour development observed for different HRP concentration; Figure S5: Comparison of spots after addition of each reagent; Figure S6: Colour development observed for each concentration of glucose solution; Figure S7: The variation of mean intensity values of all the training images (a) RGB (b) HSV (c) L*a*b (d) xyz (e) NTSC; Figure S8: The variation of overall skewness of all the training images (a) HSV (b) L*a*b (c) xyz (d) NTSC (e) RGB; Figure S9: The variation of overall kurtosis of all the training images (a) RGB (b) HSV (c) L*a*b (d) xyz (e) NTSC; Figure S10: The variation of overall values of training images obtained from GLCM (a) correlation, (b) energy, (c) contrast, (d) intensity, (e) homogeneity, and (f) entropy; Figure S11: Colour image of unknown samples for the testing of trained data; Figure S12: Shelf-life testing of spots kept at room temperature and at 4 °C for 14 days.
