# Supplementary material for: A Chemometric-Assisted Colorimetric-Based Inexpensive Paper Biosensor for Glucose Detection
Source: Biosensors (Basel). 2022 Nov 11;12(11):1008. doi: 10.3390/bios12111008 (PMC9688802; doi:10.3390/bios12111008)
Supplement: Supplementary file 1 [file biosensors-12-01008-s001.zip › biosensors-1910830-supplementary.pdf]

biosensors

Supplementary Information

Chemometric Assisted Colorimetric Based Inexpensive Paper Biosensor for Glucose Detection

Vinay Kishnani, Shrishti Kumari and Ankur Gupta*

* Correspondence: author: ankurgupta@iitj.ac.in

Table S1. EDX analysis of papers.

Types of Paper	Elemental Analysis													
	C		O		Si		Al		Ca		Mg		Cl	
Whatman No.1	45.24	52.37	54.81	47.64										
Bond Paper	40.28	48.45	55.28	49.93	0.11	0.06			4.33	1.56				
Craft Paper (200 GSM)	33.58	40.91	63.37	57.96	0.09	0.05			2.96	1.08				
Handmade/ Blotting Paper	44.62	52.36	52.57	46.32	1.54	0.77	0.59	0.31	0.69	0.24				
Paper Towel (2-Ply)	44.81	52.06	54.82	47.81					0.37	0.13				
Glossy Paper	42.70	50.45	54.85	48.65	0.16	0.08			2.28	0.81				
300GSM Card Sheet	43.89	51.05	55.99	48.90	0.02	0.01			0.10	0.04				
Practical Sheet Paper	37.14	46.32	52.59	49.23	1.67	0.89			7.19	2.69	1.41	0.87		
Ivory Sheet	43.58	51.27	53.40	47.17	1.78	0.90			0.22	0.08	1.02	0.59		
Sketch Sheet	44.68	52.21	53.21	46.67	1.28	0.64					0.83	0.48		
Drawing Sheet	43.45	50.74	55.75	48.88	0.67	0.33			0.13	0.05				
A-4 Paper (Normal Printing Paper) 67 GSM	34.76	43.12	58.24	54.24					6.22	2.31			0.78	0.33
Whatman No.4	46.12	52.90	53.88	47.10										

Weight Percentage	Atomic Percentage

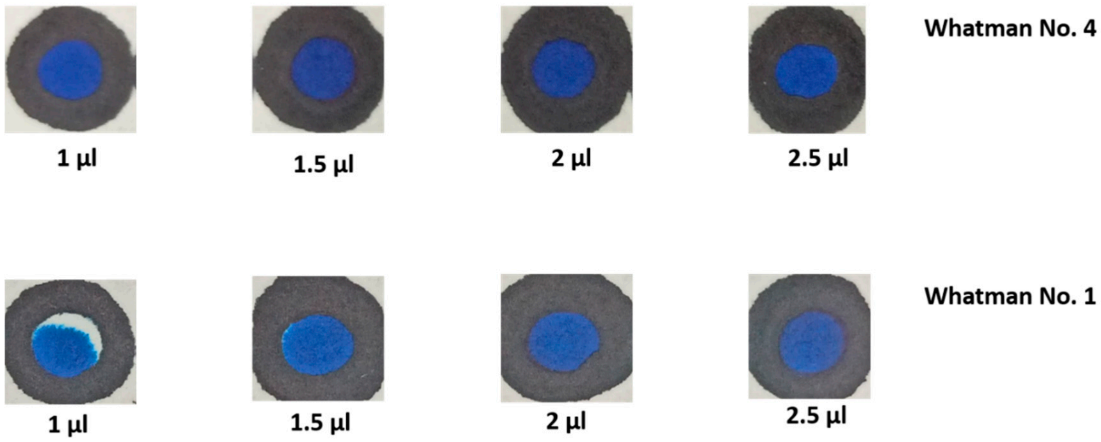

Figure S1. Sample and reagent volume optimization using MB dye solution

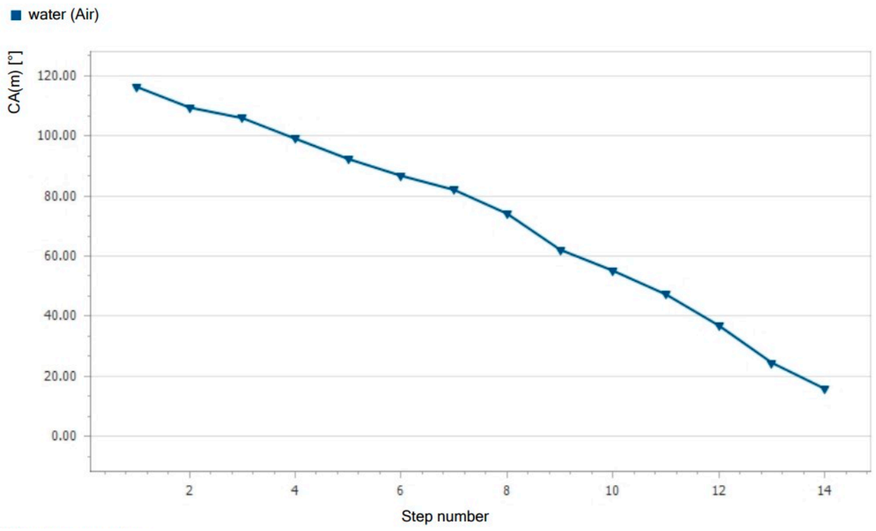

Figure S2. Contact angle measurement of single stroke coated ink over Whatman.4 paper

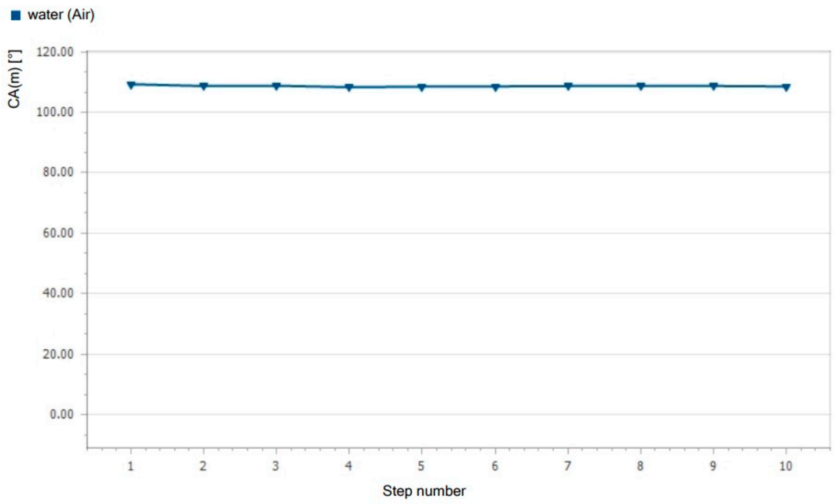

Figure S3. Contact angle measurement of double stroke coated ink over Whatman.4 paper

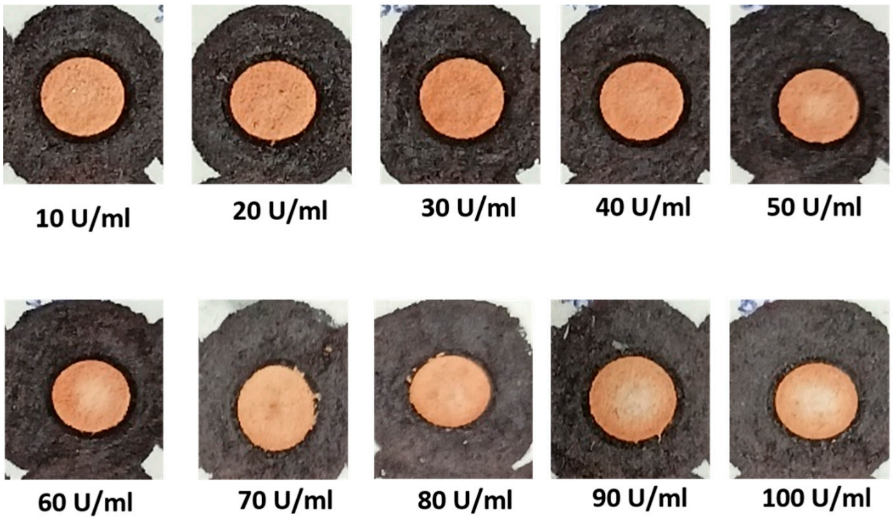

Figure S4. Colour development observed for different HRP concentration

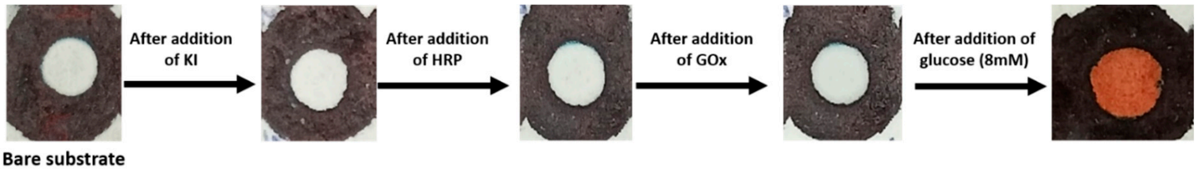

Figure S5. Comparison of spots after addition of each reagent

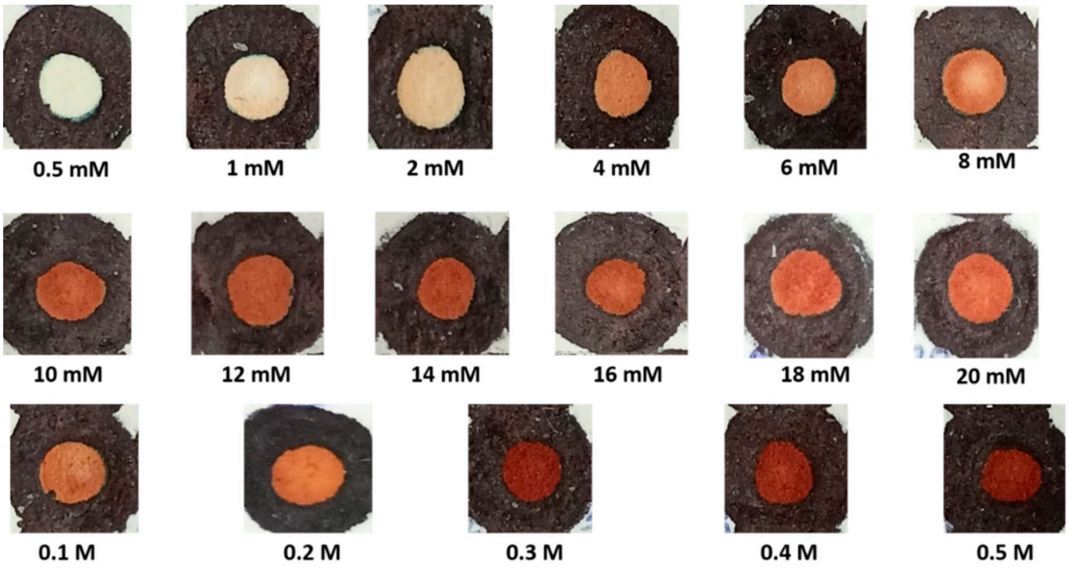

Figure S6. Colour development observed for each concentration of glucose solution

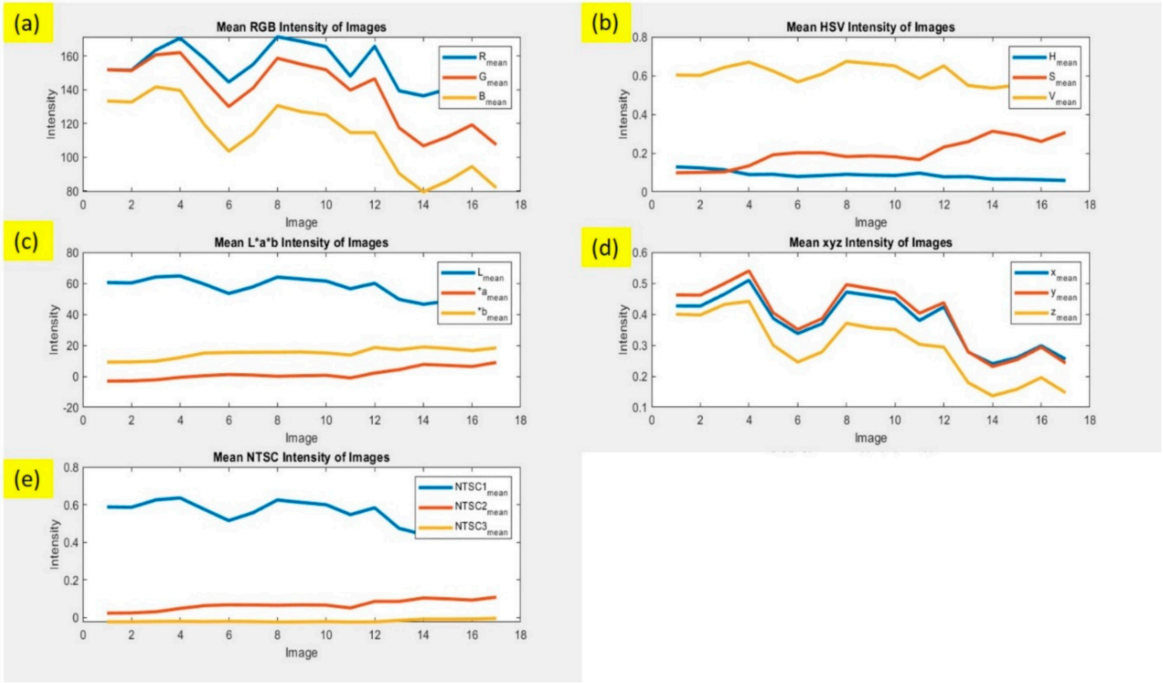

Figure S7. The variation of mean intensity values of all the training images (a) RGB (b) HSV (c) L*a*b (d) xyz (e) NTSC.

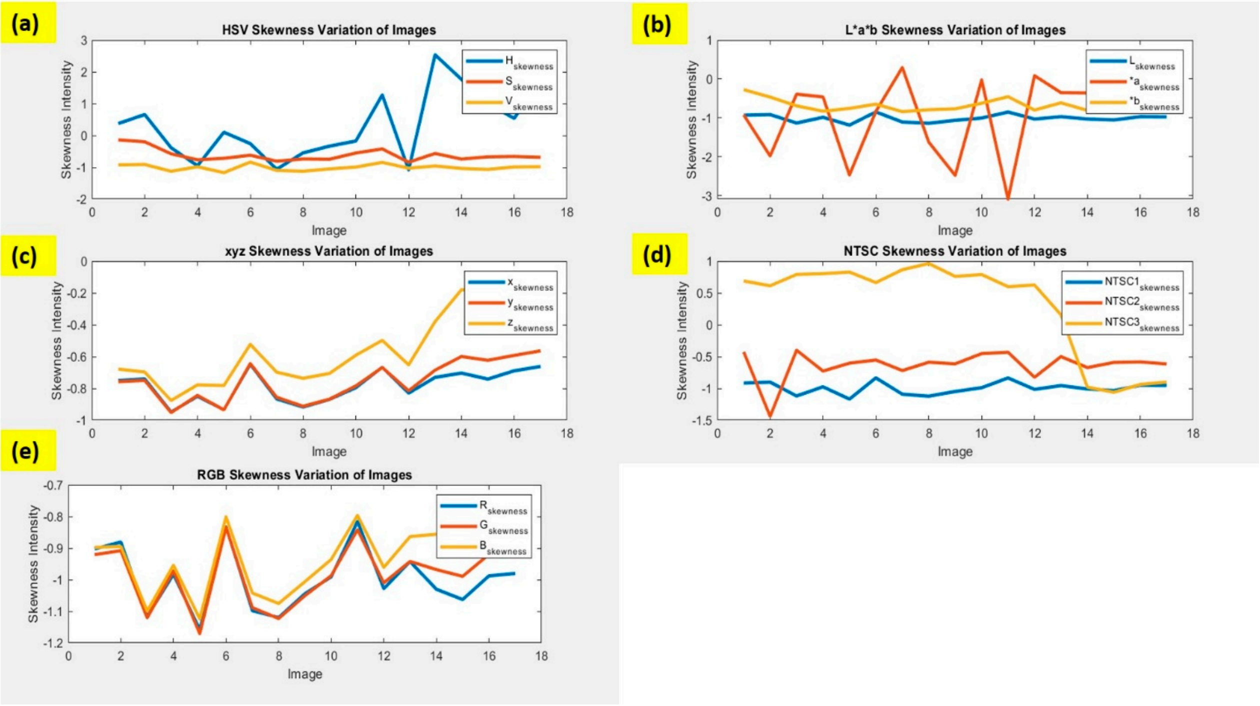

Figure S8. The variation of overall skewness of all the training images (a) HSV (b) L*a*b (c) xyz (d) NTSC (e) RGB

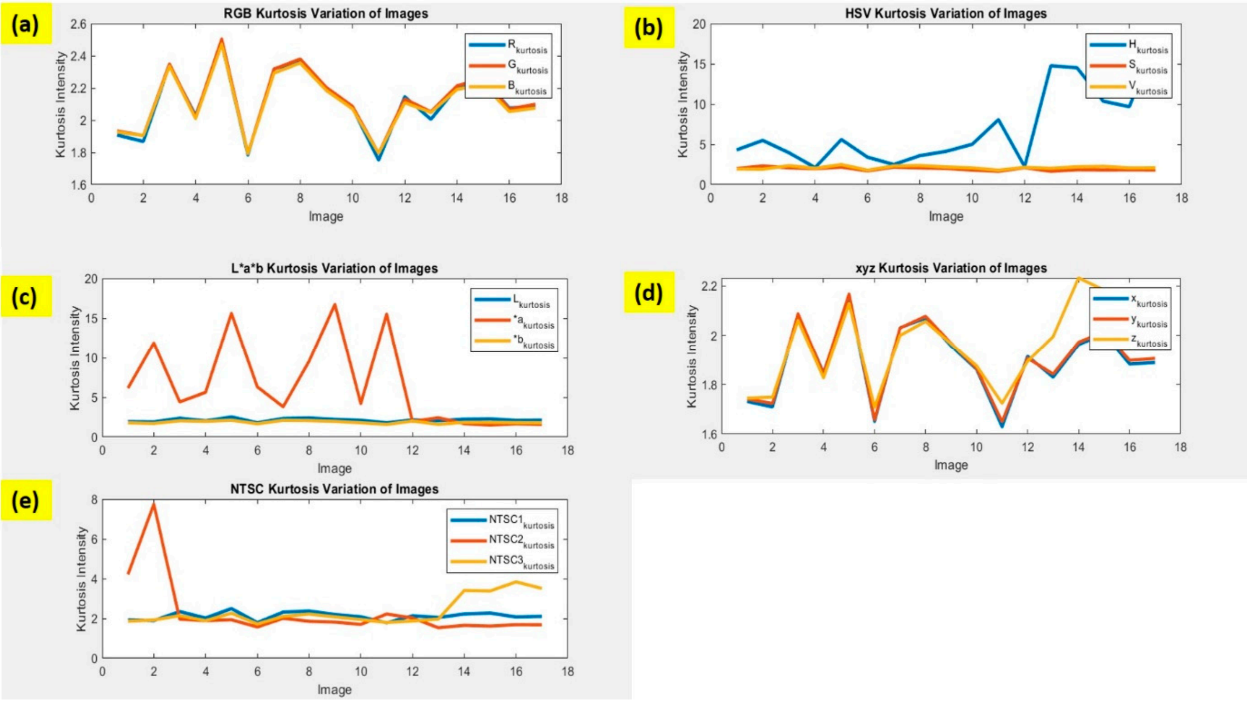

Figure S9. The variation of overall kurtosis of all the training images (a) RGB (b) HSV (c) L*a*b (d) xyz (e) NTSC.

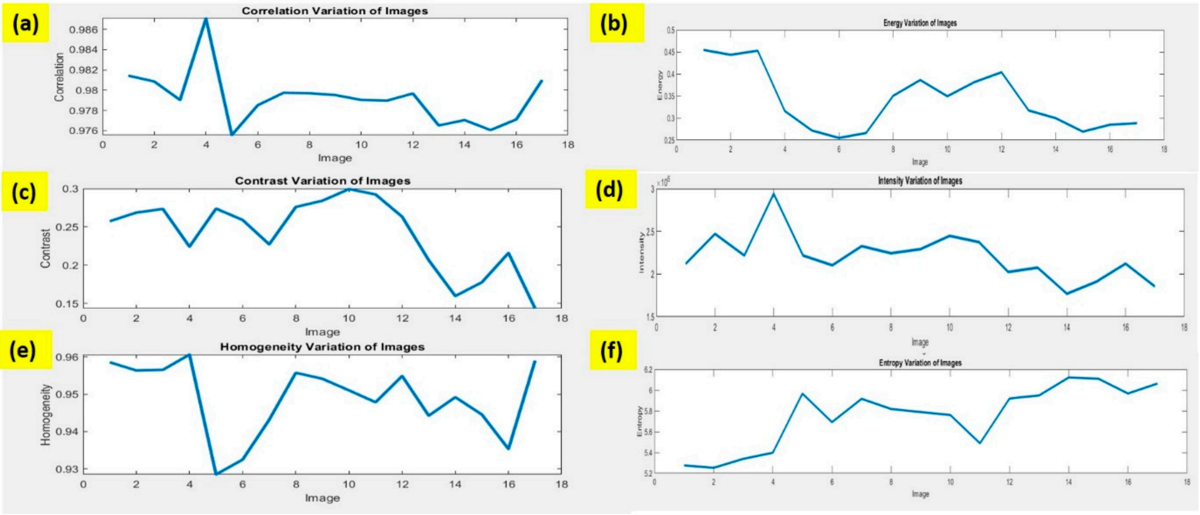

Figure S10. The variation of overall values of training images obtained from GLCM (a) correlation, (b) energy, (c) contrast, (d) intensity, (e) homogeneity, and (f) entropy.

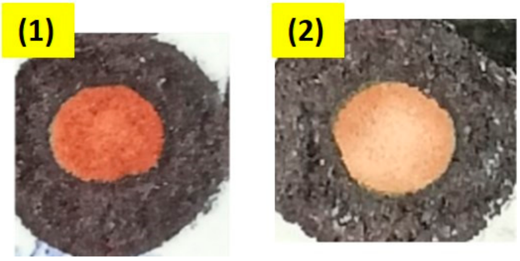

Figure S11. Colour image of unknown samples for the testing of trained data.

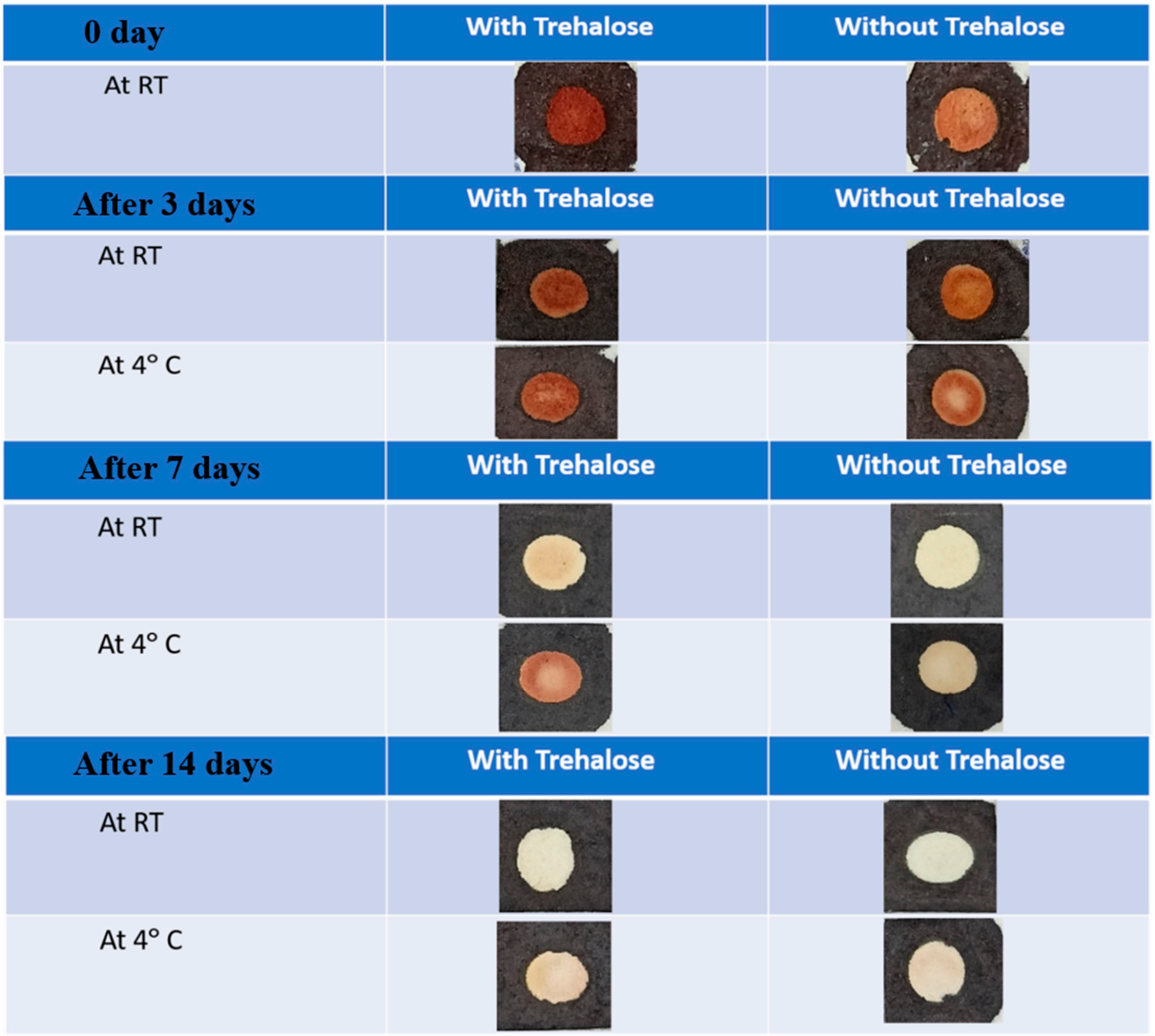

Figure S12. Shelf-life testing of spots kept at room temperature and at 4° C for 14 days
